# Supplementary material for: The use of geographic information systems (GIS) in studying mental health service delivery: A scoping review
Source: Glob Ment Health (Camb). 2025 Nov 11;12:e132. doi: 10.1017/gmh.2025.10088 (PMC12641310; doi:10.1017/gmh.2025.10088)
Supplement: Biswal et al. supplementary material [file S2054425125100885sup001.zip › S2054425125100885sup001.docx]

| **#** | **Query** |
| --- | --- |
| 1 | exp Mental Health/ |
| 2 | exp Mental Disorders/ |
| 3 | exp Stress, Psychological/ |
| 4 | exp Alzheimer Disease/ |
| 5 | exp Dementia/ |
| 6 | exp Depression/ or exp Depression, Postpartum/ |
| 7 | exp Dysthymic Disorder/ |
| 8 | exp Depressive Disorder/ or exp Depressive Disorder, Major/ |
| 9 | exp Bipolar Disorder/ |
| 10 | exp Anxiety/ or exp Anxiety Disorders/ |
| 11 | exp Psychotic Disorders/ or exp Schizophrenia/ or exp Obsessive-Compulsive Disorder/ |
| 12 | exp Adjustment Disorders/ |
| 13 | exp Mood Disorders/ |
| 14 | exp Stress Disorders, Post-Traumatic/ |
| 15 | exp Substance-Related Disorders/ |
| 16 | exp Neurotic Disorders/ |
| 17 | exp Panic Disorder/ or exp Phobic Disorders/ |
| 18 | exp Personality Disorders/ |
| 19 | exp Dissociative Disorders/ or exp Somatoform Disorders/ |
| 20 | exp Alcohol-Related Disorders/ or exp Alcoholism/ |
| 21 | exp Suicide/ or exp Suicidal Ideation/ or exp Suicide, Attempted/ |
| 22 | exp Borderline Personality Disorder/ |
| 23 | exp Self-Injurious Behavior/ |
| 24 | (mental health or mental illness* or mental health condition* or mental disorder* or psychological health).mp. [mp=title, book title, abstract, original title, name of substance word, subject heading word, floating sub-heading word, keyword heading word, organism supplementary concept word, protocol supplementary concept word, rare disease supplementary concept word, unique identifier, synonyms, population supplementary concept word, anatomy supplementary concept word] |
| 25 | (Alzheimer's or Alzheimer disorder* or Dementia).mp. [mp=title, book title, abstract, original title, name of substance word, subject heading word, floating sub-heading word, keyword heading word, organism supplementary concept word, protocol supplementary concept word, rare disease supplementary concept word, unique identifier, synonyms, population supplementary concept word, anatomy supplementary concept word] |
| 26 | (Depress* or depressive disorder* or dysthymia or dysthymic disorder*).mp. [mp=title, book title, abstract, original title, name of substance word, subject heading word, floating sub-heading word, keyword heading word, organism supplementary concept word, protocol supplementary concept word, rare disease supplementary concept word, unique identifier, synonyms, population supplementary concept word, anatomy supplementary concept word] |
| 27 | (Bipolar or bipolar disorder* or bipolar depress* or postpartum depress* or stress disorder* or post traumatic stress disorder* or PTSD).mp. [mp=title, book title, abstract, original title, name of substance word, subject heading word, floating sub-heading word, keyword heading word, organism supplementary concept word, protocol supplementary concept word, rare disease supplementary concept word, unique identifier, synonyms, population supplementary concept word, anatomy supplementary concept word] |
| 28 | (adjustment disorder* or mood disorder* or neurotic disorder*).mp. [mp=title, book title, abstract, original title, name of substance word, subject heading word, floating sub-heading word, keyword heading word, organism supplementary concept word, protocol supplementary concept word, rare disease supplementary concept word, unique identifier, synonyms, population supplementary concept word, anatomy supplementary concept word] |
| 29 | (anxiety or anxiety disorder* or panic disorder* or phobic disorder* or phobia).mp. [mp=title, book title, abstract, original title, name of substance word, subject heading word, floating sub-heading word, keyword heading word, organism supplementary concept word, protocol supplementary concept word, rare disease supplementary concept word, unique identifier, synonyms, population supplementary concept word, anatomy supplementary concept word] |
| 30 | (Schizophreni* or somatoform disorder* or personality disorder* or psychos* or psychotic disorder* or psychological trauma).mp. [mp=title, book title, abstract, original title, name of substance word, subject heading word, floating sub-heading word, keyword heading word, organism supplementary concept word, protocol supplementary concept word, rare disease supplementary concept word, unique identifier, synonyms, population supplementary concept word, anatomy supplementary concept word] |
| 31 | (Alcoholi* or alcohol-related disorder*).mp. [mp=title, book title, abstract, original title, name of substance word, subject heading word, floating sub-heading word, keyword heading word, organism supplementary concept word, protocol supplementary concept word, rare disease supplementary concept word, unique identifier, synonyms, population supplementary concept word, anatomy supplementary concept word] |
| 32 | (common mental disorder* or severe mental disorder* or common mental illness* or severe mental illness*).mp. [mp=title, book title, abstract, original title, name of substance word, subject heading word, floating sub-heading word, keyword heading word, organism supplementary concept word, protocol supplementary concept word, rare disease supplementary concept word, unique identifier, synonyms, population supplementary concept word, anatomy supplementary concept word] |
| 33 | (suicide* or suicidal ideation or suicidal* or self-harm or self-harm disorder*).mp. [mp=title, book title, abstract, original title, name of substance word, subject heading word, floating sub-heading word, keyword heading word, organism supplementary concept word, protocol supplementary concept word, rare disease supplementary concept word, unique identifier, synonyms, population supplementary concept word, anatomy supplementary concept word] |
| 34 | or/1-33 |
| 35 | exp Geographic Mapping/ |
| 36 | geographic [mapping.mp](http://mapping.mp/). [mp=title, book title, abstract, original title, name of substance word, subject heading word, floating sub-heading word, keyword heading word, organism supplementary concept word, protocol supplementary concept word, rare disease supplementary concept word, unique identifier, synonyms, population supplementary concept word, anatomy supplementary concept word] |
| 37 | (GIS or ArcGIS or QGIS).mp. [mp=title, book title, abstract, original title, name of substance word, subject heading word, floating sub-heading word, keyword heading word, organism supplementary concept word, protocol supplementary concept word, rare disease supplementary concept word, unique identifier, synonyms, population supplementary concept word, anatomy supplementary concept word] |
| 38 | spatial analys*.mp. [mp=title, book title, abstract, original title, name of substance word, subject heading word, floating sub-heading word, keyword heading word, organism supplementary concept word, protocol supplementary concept word, rare disease supplementary concept word, unique identifier, synonyms, population supplementary concept word, anatomy supplementary concept word] |
| 39 | exp Spatial Analysis/ |
| 40 | spatial [epidemiology.mp](http://epidemiology.mp/). [mp=title, book title, abstract, original title, name of substance word, subject heading word, floating sub-heading word, keyword heading word, organism supplementary concept word, protocol supplementary concept word, rare disease supplementary concept word, unique identifier, synonyms, population supplementary concept word, anatomy supplementary concept word] |
| 41 | (geospatial analys* or geographic analys*).mp. [mp=title, book title, abstract, original title, name of substance word, subject heading word, floating sub-heading word, keyword heading word, organism supplementary concept word, protocol supplementary concept word, rare disease supplementary concept word, unique identifier, synonyms, population supplementary concept word, anatomy supplementary concept word] |
| 42 | exp Geographic Information Systems/ |
| 43 | geographic information system*.mp. [mp=title, book title, abstract, original title, name of substance word, subject heading word, floating sub-heading word, keyword heading word, organism supplementary concept word, protocol supplementary concept word, rare disease supplementary concept word, unique identifier, synonyms, population supplementary concept word, anatomy supplementary concept word] |
| 44 | geographic information science*.mp. [mp=title, book title, abstract, original title, name of substance word, subject heading word, floating sub-heading word, keyword heading word, organism supplementary concept word, protocol supplementary concept word, rare disease supplementary concept word, unique identifier, synonyms, population supplementary concept word, anatomy supplementary concept word] |
| 45 | or/35-44 |
| 46 | 34 and 45 |
| 47 | limit 46 to (english language and yr="1981 - 2024") |
