# Supplementary material for: The use of geographic information systems (GIS) in studying mental health service delivery: A scoping review
Source: Glob Ment Health (Camb). 2025 Nov 11;12:e132. doi: 10.1017/gmh.2025.10088 (PMC12641310; doi:10.1017/gmh.2025.10088)
Supplement: Biswal et al. supplementary material [file S2054425125100885sup001.zip › S2054425125100885sup002.pdf]

Geographic Information Systems (GIS) in understanding mental health service delivery: A Scoping Review Protocol

Bijayalaxmi Biswal[1], Luanna Fernandes[1], Arista Jhanjee[2], Yashi Gandhi[1], Abhijit Nadkarni[1,3]

[1] Addictions and related-Research Group, Sangath, Goa, India

[2] Harvard T.H. Chan School of Public Health, Harvard University, USA

[3] Department of Population Health, London School of Hygiene & Tropical Medicine, London, United Kingdom

Abstract

Introduction: The use of Geographic Information Systems (GIS) to study availability, accessibility, and utilization of healthcare services for mental health problems is still unexplored. We intend to define the existing landscape in order to fully comprehend the possibilities of using GIS in the field of mental health research, implementation science, mental healthcare planning and service delivery.

Objective: The objective of the review is to identify the scope and use of GIS in understanding accessibility, utilization, and availability of mental health services, to explore any gaps and future possibilities in the applications of GIS.

Inclusion Criteria: The current scoping review will include all population groups across the life course. Studies published in English language will be included. Studies will be included if they were published in the year 1981 (the year when GIS technology first became commercially available to the public) or later. Primary and secondary research papers of any design and methodology will be included. We will exclude reviews, commentaries, and opinion pieces. The concepts that will be searched are GIS and mental health conditions. Only studies focusing on one or more of the three dimensions of healthcare service delivery (utilization, accessibility and availability) will be included.

Methods: We will follow a scoping review methodology (Arksey & O'Malley, 2005; Peters et al., 2015). Embase, Medline, APA PsycINFO, CINAHL, CENTRAL , Global Health and Web of Science will be searched for studies in English Language, from date of public availability of GIS (1981) to date of search (2023). In addition, backward and forward citation chaining will be conducted to supplement the electronic database search. Data on applications of GIS, GIS data collection tools used, definitions as well as measures for the three dimensions of service delivery, and mental health services examined will be extracted.

Results: Results will be conceptually mapped and narratively summarised to capture the various ways in which GIS has been applied in the field of mental health research and service delivery.

Keywords
Scoping Review, Mental Health, Geographic Information Systems, Service Delivery, Accessibility, Availability, Utilization

Introduction

Geographic Information Systems (GIS) have been widely used in the field of public health, especially for understanding the spatial organization of healthcare, studying utilization patterns, and mapping the availability of healthcare services (Graves, 2008; Higgs, 2004, 2009; McLafferty, 2003). However, the use of GIS to study delivery of mental healthcare services is still unexplored. This has not only precluded a comprehensive understanding of the full potential of GIS in mental health research, implementation science, health planning and service delivery, but also limited the possibilities of its usage. This review aims to address this gap by mapping the scope and type of GIS usage in studying three dimensions of mental health service delivery, across all geographical locations, settings and populations. These three dimensions are: accessibility, availability, and utilization.

According to the WHO Health Systems Framework, service delivery is one of the six core components or "building blocks" of a health system (WHO, 2017). Parameters considered by them for monitoring a healthcare service delivery system include:

i. Accessibility: geographic accessibility or spatial accessibility, in terms of commuting time spent and distance traversed to reach healthcare services (Penchansky, 1981). For example, distribution of health facilities offering specific services per 10,000 population
ii. Utilization: quantification or description of the use of healthcare services by people to study trends, patterns, variations or for other objectives (Carrasquillo, 2013). For example, number of outpatient department visits per 10,000 population per year and,
iii. Availability of services: physical presence of services and encompasses health infrastructure, core health personnel and aspects of service utilization (WHO, 2015). For example, proportion of health facilities offering specific services.

For this review, we consider these three dimensions of service delivery as they can also be spatially analysed, hence providing opportunity for GIS applications.

While we limit the definition of "accessibility" to its geographic aspect, we are aware that it's a broader concept determined by other factors that affect one's uptake of healthcare (Andersen and Newman, 1973). We use "utilization" as a separate concept to capture any studies which might highlight the direct or indirect use of GIS in analysing any other aspects of service delivery, especially non-spatial (eg: acceptability or affordability of services). We also anticipate that

exploring the concept "utilization" can help us discover studies that have used GIS to assess inequity or disparities in care and explain variations in healthcare use. As we are uncertain about the breadth and diversity of evidence and do not have extensive literature on the subject for reference, the exploratory and flexible nature of scoping reviews was considered an ideal methodology for the project.

No current or underway systematic reviews or scoping reviews on the topic were identified after conducting a search on PROSPERO and Open Science Framework (OSF). A preliminary search of MEDLINE, Embase and APA PsycINFO was done to identity reviews done on the topic in the past. An integrative review conducted in 2019 (Emt-B & Neff, 2019) reviewed GIS applications that were used to study accessibility of mental healthcare services but limited its scope only to services provided for serious mental illnesses.

This scoping review aims to map the scope of employing GIS in understanding mental health service delivery, across all contexts and populations, in order to understand gaps and future possibilities. We aim to provide an overview of the literature while highlighting key differences in usage (if any) based on concept definition, study design and setting.

Review Question

What are the ways in which GIS has been applied/used to study accessibility, utilization and availability of mental health services?

Eligibility criteria

Population: The current scoping review includes all groups across the life course. There is no specified population.

Concept: Broadly, the scoping review aims to explore the evidence base on:
(1) GIS and its various uses, and (2) mental health conditions

Table 1 contains search terms used for each concept/keyword. We will include any mental health condition that is diagnosed using one of the following: a) Diagnostic and Statistical Manual of Mental Disorders Fifth edition (DSM-V) and the International Classification for Diseases- Eleventh Revision (ICD- 11) diagnostic criteria; b) Positive screen on a validated rating scale or standardized diagnostic instrument designed according to the DSM/ICD diagnostic criteria (i.e., PHQ-9, GAD-7); c) Clinician diagnosis.

We will include any study that uses a GIS software to analyse geographical data. We will exclude studies solely using Global Positioning Systems (GPS) because it is fundamentally different from GIS and does not analyse geographical data. We have included names of popular GIS software (eg: QGIS, ArcGIS) as alternate keywords to widen the search but also intend to capture any local innovations in the area, and observe differences in GIS data collection tools used in high income and low-and-middle income contexts.

Only studies focusing on service delivery (utilization, accessibility, and availability) of healthcare services will be included. Healthcare services is defined to cover evidence-based primary, secondary and tertiary healthcare as well as community mental health services but not interventions which are not traditionally categorised as healthcare (eg: social interventions that improve mental health). We define utilization as quantification or description of the use of healthcare services by people to study trends, patterns, variations or for other objectives (Carrasquillo, 2013). Accessibility is defined primarily as geographic accessibility or spatial accessibility, in terms commuting time spent and distance traversed to reach healthcare services (Penchansky, 1981). Service availability refers to the physical presence of services and encompasses health infrastructure, core health personnel and aspects of service utilization (WHO, 2015).

Context: Studies published in English language from 1981 up until July 2023 will be considered

Types of Sources: Primary and secondary research papers of any design and methodology (including quantitative and qualitative design) will be included if they meet the inclusion criteria. Both experimental and quasi-experimental study designs including randomized controlled trials, non-randomized controlled trials, analytical observational studies including prospective and retrospective cohort studies, case-control studies and analytical cross-sectional studies will be considered for inclusion. This review will also consider descriptive observational study designs including case reports, case series, and descriptive cross-sectional studies for inclusion. We will exclude reviews, commentaries, and opinion pieces.

Table 1: Keywords and Search Terms

Construct	Search Terms
Mental health conditions	"mental disorder" OR "mental health condition" OR "mental health" OR "psychological health" OR "Alzheimer's" OR "Dementia" OR "depression" OR "depressive disorder" OR "depression" OR "dysthymia" OR "postpartum" OR "bipolar" OR "adjustment disorder" OR "mood disorder" OR "neurotic disorder" OR "stress disorder" OR "anxiety disorder" OR "schizophrenia" OR "somatoform disorder" OR "panic disorder" OR "Phobic disorder" OR "personality disorders" OR "psychological trauma" OR "psychotic disorder" OR "psychosis" OR "substance-related disorders" OR "alcoholism" OR "common mental disorder" OR "severe mental disorder" OR "suicidality" OR "suicidal ideation" OR "self-harm disorder" OR "bipolar disorder" OR "stress disorders"
GIS	"GIS" OR "Geographic information systems" OR "Geographic mapping" OR "Spatial analysis" OR "Geospatial analysis" OR "Geographic information science" OR "Geographic analysis" OR "Spatial epidemiology"

Methods

The project will employ a scoping review methodology, which aims to define and establish the scope of existing literature and map the currently available evidence (Arksey & O'Malley, 2005; Peters et al., 2015). Scoping reviews intend to determine the coverage of a specific topic and provide a comprehensive overview of the literature, as opposed to systematic reviews which aim to answer very specific questions. Scoping reviews also differ from traditional literature reviews by adhering to a predetermined protocol, conducting a systematic and thorough search, and striving for transparency and reproducibility (Munn et al., 2018).

The review will be conducted in accordance with the Joanna Briggs Institute Methodology for Scoping Reviews (2020) and will incorporate the Preferred Reporting Items for Systematic Reviews and Meta-Analysis (PRISMA) extension for Scoping Reviews Checklist (Tricco et al., 2018).

Search Strategy

Eligible studies will be identified through a systematic search of health-related databases. The systematic search will use search terms, their synonyms, and alternative spellings, combined using Boolean operators: AND for different concepts and OR for synonyms.

Electronic database search: The following databases will be searched: Embase, Medline, APA PsycINFO, CINAHL, CENTRAL, Global Health and Web of Science.

Other sources: Backward and forward citation chaining will be conducted to supplement the electronic database search.

Study Selection

Following the search, all results from different databases will be imported into EndNote. After removing duplicate records on EndNote, studies will be uploaded to Covidence, for further deduplication (automatic and manual) and screening process. All papers will be screened independently by both the reviewers. The interrater reliability (IRR) will be calculated as a Kappa statistic and an agreement percentage above 80% will be regarded as acceptable. Any conflicts in judgment will be resolved through discussion until a consensus is reached. If no consensus is reached between the two reviewers, a third reviewer will resolve conflicts.

Full texts of studies that can be potentially included will be retrieved and will be independently reviewed by two researchers. Justification for exclusion will be recorded in Covidence. In case of conflicts during full-text screening, a third reviewer will resolve conflicts.

Charting the Results

Pre-piloted data collection forms on MS Excel will be used to extract relevant data from each included study by two members of our team independently. The process is guided by recommendations from '*Guidance for conducting systematic scoping reviews*' (Peters et al., 2015). Below is an overview of the data extraction form. Due to the exploratory nature of this scoping review, the data extraction form may be modified and revised if necessary to ensure all relevant data is included. The final data extraction form will be detailed in the scoping review.

1. Publication (e.g., authors, publication year etc.)
2. Location of application (e.g., country, setting etc.)
3. Research question/ aim of the study
4. Study design (e.g., intervention study, observational study etc.)
5. Study methodology (eg: quantitative, qualitative, mixed)
6. Study population (eg: Sample size and characteristics etc)
7. Service delivery dimension explored (accessibility or availability or utilization of healthcare services)
8. Definition of accessibility used in the study
9. Measures of accessibility (e.g., travel distance, travel time)
10. Mental health disorder and service examined for accessibility
11. Definition of utilization used in the study
12. Measures of utilization (e.g., number of visits, frequency of use)
13. Mental health disorder and service examined for utilization
14. Definition of availability used in the study
15. Measures of availability (e.g., number of mental health facilities, distribution of services, density of service)
16. Mental health disorder and service examined for availability
17. Types of GIS data collection tools, sources or software utilized
18. Type of GIS application/use (e.g., mapping, spatial analysis)
19. Other relevant information TBC following screening of studies

Any disagreements that arise between the reviewers during extraction, will be resolved through discussion, or with the help of an additional reviewer. Authors of papers will be contacted to request missing or additional data, where required.

Data Quality Assessment

In line with guidelines for scoping reviews (Peters, 2015), we will not be conducting quality assessments of the included studies.

Data Analysis and Presentation

To effectively summarise the findings in accordance with the objectives of the review, we will utilise a narrative synthesis. The Preferred Reporting Items for Systematic Reviews and MetaAnalysis (PRISMA) extension for Scoping Reviews Checklist and Flow Diagram will be used to report the search process. The results will be presented in conceptual categories aligned with the type of GIS use. Visual representation, such as a data "map" or tabular format, will be

considered based on which method best illustrates the results in relation to the aims and research questions. A key focus of our review is to identify gaps in the existing literature and provide recommendations for future research, including potential areas for future systematic reviews, context-specific investigations, and unexplored possibilities. Depending on availability of data, we will develop a framework or conceptual mapping of the identified applications/uses.

Conflicts of Interest

The authors declare that they have no conflict of interest in this project.

Funding

This study is a part of the IMPRESS (IMPlementation of evidence-based facility and community interventions to reduce the treatment gap for depRESSion) trial which aims to assess the effectiveness and cost-effectiveness of a community intervention in enhancing access to care and improving clinical outcomes for depression. The trial has been funded through a grant from the National Institute of Mental Health (NIMH), USA (Grant number R01MH115504).

References

Andersen, R.M., & Newman, J.F. (1973). Societal and individual determinants of medical care utilization in the United States. The Milbank Memorial Fund quarterly. *Health and society*, 51 1, 95-124 .

Arksey, H., & O'Malley, L. (2005). Scoping studies: Towards a methodological framework. *International Journal of Social Research Methodology: Theory and Practice*, *8*(1), 19–32. https://doi.org/10.1080/1364557032000119616

Atun, R., Aydin, S., Chakraborty, S., Sümer, S., Aran, M., Gürol, I., Nazlioğlu, S., Özgülcü, Ş., Aydoğan, Ü., Ayar, B., Dilmen, U., & Akdağ, R. (2013). Universal health coverage in Turkey: Enhancement of equity. In *The Lancet* (Vol. 382, Issue 9886, pp. 65–99). Elsevier B.V. https://doi.org/10.1016/S0140-6736(13)61051-X

Carrasquillo, O. (2013). Health care utilization. *Encyclopedia of behavioral medicine*, 909-910.

Emt-B, M. S. D. A., & Neff, D. L. (2019). Mental Health Care Access Using Geographic Information Systems: An Integrative Review. *Issues in Mental Health Nursing*, 41(2), 113–121. https://doi.org/10.1080/01612840.2019.1646363

Graves, B. A. (2008). *Integrative Literature Review: A Review of Literature Related to Geographical Information Systems, Healthcare Access, and Health Outcomes*.

Higgs, G. (2004). A Literature Review of the Use of GIS-Based Measures of Access to Health Care Services. In *Health Services & Outcomes Research Methodology* (Vol. 5).

Higgs, G. (2009). The role of GIS for health utilization studies: Literature review. In *Health Services and Outcomes Research Methodology* (Vol. 9, Issue 2, pp. 84–99). https://doi.org/10.1007/s10742-009-0046-2

McLafferty, S. L. (2003). GIS and health care. *Annual Review of Public Health*, *24*, 25–42. https://doi.org/10.1146/annurev.publhealth.24.012902.141012

Munn, Z., Peters, M. D. J., Stern, C., Tufanaru, C., McArthur, A., & Aromataris, E. (2018). Systematic review or scoping review? Guidance for authors when choosing between a systematic or scoping review approach. *BMC Medical Research Methodology*, *18*(1). https://doi.org/10.1186/s12874-018-0611-x

Peters, M. D. J., Godfrey, C. M., Khalil, H., McInerney, P., Parker, D., & Soares, C. B. (2015). Guidance for conducting systematic scoping reviews. *International Journal of Evidence-Based Healthcare*, *13*(3), 141–146. https://doi.org/10.1097/XEB.0000000000000050

Penchansky R, Thomas JW. The concept of access: definition and relationship to consumer satisfaction. *Med Care. 1981 Feb;19(2)*:127-40. doi: 10.1097/00005650-198102000-00001. PMID: 7206846.

Tricco, A. C., Lillie, E., Zarin, W., O'Brien, K. K., Colquhoun, H., Levac, D., Moher, D., Peters, M. D. J., Horsley, T., Weeks, L., Hempel, S., Akl, E. A., Chang, C., McGowan, J., Stewart, L., Hartling, L., Aldcroft, A., Wilson, M. G., Garritty, C., … Straus, S. E. (2018). PRISMA extension for scoping reviews (PRISMA-ScR): Checklist and explanation. In *Annals of Internal Medicine* (Vol. 169, Issue 7, pp. 467–473). American College of Physicians. https://doi.org/10.7326/M18-0850

World Health Organization. (2015). *Service Availability and Readiness Assessment (SARA) | Reference Manual, Version 2.2*. World Health Organization.

World Health Organization. (2010). *Monitoring the building blocks of health systems*. World Health Organization.
