# Supplementary material for: The use of geographic information systems (GIS) in studying mental health service delivery: A scoping review
Source: Glob Ment Health (Camb). 2025 Nov 11;12:e132. doi: 10.1017/gmh.2025.10088 (PMC12641310; doi:10.1017/gmh.2025.10088)
Supplement: Biswal et al. supplementary material [file S2054425125100885sup001.zip › S2054425125100885sup003.docx]

**Appendix 3: Studies excluded at full-text screening**

|  | **Citation** | **Reason for exclusion** |
| --- | --- | --- |
| 1. | Abraham AJ, Andrews CM, Yingling ME, Shannon J. Geographic Disparities in Availability of Opioid Use Disorder Treatment for Medicaid Enrollees. Health Services Research. 2018 Feb;53(1):389–404. | GIS not used |
| 2. | Pompeii LA, Lipscomb HJ, Dement JM. Predictors of lost time from work among nursing personnel who sought treatment for back pain. Work. 2010;37(3):285–95. | GIS not used |
| 3. | Hsu ZS, Warnick JA, Harkins TR, Sylvester BE, et al. An Analysis of Patterns of Distribution of Buprenorphine in the United States using ARCOS, Medicaid, and Medicare Databases [Internet]. Addiction Medicine; 2022. Available from: <http://medrxiv.org/lookup/doi/10.1101/2022.08.22.22279092> | GIS not used |
| 4. | Chen TC, Chen LC, Kerry M, Knaggs RD. Prescription opioids: Regional variation and socioeconomic status – evidence from primary care in England. International Journal of Drug Policy. 2019 Feb;64:87–94. | GIS not used |
| 5. | Krawczyk N, Jent V, Hadland SE, Cerdá M. Utilization of Medications for Opioid Use Disorder Across US States: Relationship to Treatment Availability and Overdose Mortality. Journal of Addiction Medicine. 2022 Jan;16(1):114–7. | GIS not used |
| 6. | Lai JT, Chapman BP, Carreiro SP, Babu KM, Boyer EW, Chai PR. Understanding Naloxone Uptake from an Emergency Department Distribution Program Using a Low-Energy Bluetooth Real-time Location System. J Med Toxicol. 2020 Oct;16(4):405–15. | GIS not used |
| 7. | He FT, Lundy De La Cruz N, Olson D, Lim S, Levanon Seligson A, Hall G, et al. Temporal and Spatial Patterns in Utilization of Mental Health Services During and After Hurricane Sandy: Emergency Department and Inpatient Hospitalizations in New York City. Disaster med public health prep. 2016 Jun;10(3):512–7. | GIS not used |
| 8. | Penfold RB, Wang W, Pajer K, Strange B, Kelleher KJ. Spatio‐temporal clusters of new psychotropic medications among Michigan children insured by Medicaid. Pharmacoepidemiology and Drug. 2009 Jul;18(7):531–9. | GIS not used |
| 9. | Lew D, Rigdon SE. Mapping rates of inpatient hospitalizations related to mental disorders in the state of Missouri: A conditional autoregressive model with zip code-level data. Spatial and Spatio-temporal Epidemiology. 2019 Feb;28:24–32. | GIS not used |
| 10. | Mair C, Sumetsky N, Kranich C, Freisthler B. Availability of medical cannabis dispensaries and cannabis abuse/dependence‐related hospitalizations in California. Addiction. 2021 Jul;116(7):1908–13. | GIS not used |
| 11. | Joudrey PJ, Kolak M, Lin Q, Paykin S, Anguiano V, Wang EA. Assessment of Community-Level Vulnerability and Access to Medications for Opioid Use Disorder. JAMA Netw Open. 2022 Apr 19;5(4):e227028. | GIS not used |
| 12. | Hirchak KA, Murphy SM. Assessing Differences in the Availability of Opioid Addiction Therapy Options: Rural Versus Urban and American Indian Reservation Versus Nonreservation. The Journal of Rural Health. 2017 Jan;33(1):102–9. | GIS not used |
| 13. | Hepler S, McKnight E, Bonny A, Kline D. A Latent Spatial Factor Approach for Synthesizing Opioid-Associated Deaths and Treatment Admissions in Ohio Counties. Epidemiology. 2019 May;30(3):365–70. | GIS not used |
| 14. | Pollack HA, Lee F, Paykin S, Aguilera JAR. Critical access medication for opioid use disorder (MOUD) treatment facilities in the continental United States. Drug and Alcohol Dependence Reports. 2023 Mar;6:100130. | GIS not used |
| 15. | Mathis WS, Woods S, Srihari V. Blind Spots: Spatial analytics can identify nonrandom geographic variation in first episode psychosis program enrollments. Early Intervention Psych. 2018 Dec;12(6):1229–34. | GIS not used |
| 16. | Cao Y, Stewart K, Wish E, Artigiani E, Sorg MH. Determining spatial access to opioid use disorder treatment and emergency medical services in New Hampshire. Journal of Substance Abuse Treatment. 2019 Jun;101:55–66. | GIS not used |
| 17. | De Marco M, Brandi AL, Bieger A, Krug B, Camozzato A, Picon PD, et al. Disparity in the use of Alzheimer’s disease treatment in Southern Brazil. Sci Rep. 2023 Jun 12;13(1):9555. | GIS not used |
| 18. | Rosychuk RJ, Johnson DW, Urichuk L, Dong K, Newton AS. Does emergency department use and post-visit physician care cluster geographically and temporally for adolescents who self-harm? A population-based 9-year retrospective cohort study from Alberta, Canada. BMC Psychiatry. 2016 Dec;16(1):229. | GIS not used |
| 19. | Thiessen M, Cui Q, Hu XJ, Rosychuk RJ. Exploring spatio-temporal patterns in mental health related emergency department use from children and adolescents. Spatial and Spatio-temporal Epidemiology. 2020 Aug;34:100358. | GIS not used |
| 20. | Rosychuk RJ, Newton AS, Niu X, Urichuk L. Space and time clustering of adolescents’ emergency department use and post-visit physician care for mood disorders in Alberta, Canada: A population-based 9-year retrospective study. CJPH. 2014 Nov 6;106(2):e10–6. | GIS not used |
| 21. | Bacolod M, Heissel J, Shen YC. Spatial Analysis of Access to Psychiatrists for US Military Personnel and Their Families. JAMA Netw Open. 2023 Jan 3;6(1):e2249314. | GIS not used |
| 22. | Maas C, Salinas-Perez JA, Bagheri N, Rosenberg S, Campos W, Gillespie JA, et al. A spatial analysis of referrals to a primary mental health programme in Western Sydney from 2012 to 2015. Geospat Health [Internet]. 2019 Nov 6 [cited 2025 Oct 10];14(2). Available from: <https://geospatialhealth.net/index.php/gh/article/view/773> | Does not focus on service delivery |
| 23. | Nolen S, Trinidad AJ, Jordan AE, Green TC, Jalali A, Murphy SM, et al. Racial/ethnic differences in receipt of naloxone distributed by opioid overdose prevention programs in New York City. Harm Reduct J. 2023 Oct 18;20(1):152. | Does not focus on service delivery |
| 24. | Young SG, Hayes CJ, Aram J, Tait MA. Doctor hopping and doctor shopping for prescription opioids associated with increased odds of high‐risk use. Pharmacoepidemiology and Drug. 2019 Aug;28(8):1117–24. | Does not focus on service delivery |
| 25. | Payne B, Gainey R. Mapping Elder Mistreatment Cases: Interactions Between Mistreatment, Dementia, Service Utilization, Access to Services, and Disadvantage. J of Human Behavior in the Social Env. 2009 Dec;19(8):1025–41. | Does not focus on service delivery |
| 26. | Polling C, Bakolis I, Hotopf M, Hatch SL. Differences in hospital admissions practices following self-harm and their influence on population-level comparisons of self-harm rates in South London: an observational study. BMJ Open. 2019 Oct;9(10):e032906. | Does not focus on service delivery |
| 27. | Cuadros DF, Tomita A, Vandormael A, Slotow R, Burns JK, Tanser F. Spatial structure of depression in South Africa: A longitudinal panel survey of a nationally representative sample of households. Sci Rep. 2019 Jan 30;9(1):979. | Does not focus on service delivery |
| 28. | Hernandez A, Branscum AJ, Li J, MacKinnon NJ, Hincapie AL, Cuadros DF. Epidemiological and geospatial profile of the prescription opioid crisis in Ohio, United States. Sci Rep. 2020 Mar 9;10(1):4341. | Does not focus on service delivery |
| 29. | Walker LR, Mason M, Cheung I. Adolescent Substance Use and Abuse Prevention and Treatment: Primary Care Strategies Involving Social Networks and the Geography of Risk and Protection. J Clin Psychol Med Settings. 2006 Jun;13(2):126–34. | Does not focus on service delivery |
| 30. | Hobbs M, Milfont TL, Marek L, Yogeeswaran K, Sibley CG. The environment an adult resides within is associated with their health behaviours, and their mental and physical health outcomes: a nationwide geospatial study. Social Science & Medicine. 2022 May;301:114801. | Does not focus on service delivery |
| 31. | Sadler RC, Furr-Holden D. The epidemiology of opioid overdose in Flint and Genesee County, Michigan: Implications for public health practice and intervention. Drug and Alcohol Dependence. 2019 Nov;204:107560. | Does not focus on service delivery |
| 32. | Stahler GJ, Mennis J, Cotlar R, Baron DA. The Influence of Neighborhood Environment on Treatment Continuity and Rehospitalization in Dually Diagnosed Patients Discharged From Acute Inpatient Care. AJP. 2009 Nov;166(11):1258–68. | Does not focus on service delivery |
| 33. | Gruebner O, Lowe SR, Sampson L, Galea S. The geography of post-disaster mental health: spatial patterning of psychological vulnerability and resilience factors in New York City after Hurricane Sandy. Int J Health Geogr. 2015 Dec;14(1):16. | Does not focus on service delivery |
| 34. | Vannier C, Campbell M, Kingham S. Pathways to urban health and well-being: measuring and modelling of community services’ in a medium size city. Geospat Health [Internet]. 2020 Jun 19;15(1). Available from: <https://www.geospatialhealth.net/index.php/gh/article/view/808> | Does not focus on mental health |
| 35. | Furr-Holden CDM, Milam AJ, Nesoff ED, Garoon J, Smart MJ, Duncan A, et al. Triangulating Syndemic Services and Drug Treatment Policy: Improving Drug Treatment Portal Locations in Baltimore City. Progress in Community Health Partnerships. 2016;10(2):319–27. | Does not focus on mental health |
| 36. | Reshadat S, Zangeneh A, Saeidi S, Teimouri R, Yigitcanlar T. Measures of spatial accessibility to health centers: investigating urban and rural disparities in Kermanshah, Iran. J Public Health (Berl). 2019 Aug;27(4):519–29. | Does not focus on mental health |
| 37. | Pierse T, Keogh F, O’Shea E, Cullinan J. Geographic availability and accessibility of day care services for people with dementia in Ireland. BMC Health Serv Res. 2020 Dec;20(1):476. | Does not focus on healthcare services |
| 38. | Leung M, Chow CB, Ip PKP, Yip SF Paul. Geographical accessibility of community social services and incidence of self-harm. Spatial and Spatio-temporal Epidemiology. 2020 Jun;33:100334. | Does not focus on healthcare services |
| 39. | Hooley C, Salvo D, Brown DS, Brookman-Frazee L, Lau AS, Brownson RC, et al. Scaling-up Child and Youth Mental Health Services: Assessing Coverage of a County-Wide Prevention and Early Intervention Initiative During One Fiscal Year. Adm Policy Ment Health. 2023 Jan;50(1):17–32. | Not focused on the three dimensions of service delivery |
| 40. | Garrido-Cumbrera M, Almenara-Barrios J, López-Lara E, Peralta-Sáez JL, García-Gutierrez JC, Salvador-Carulla L. Development and spatial representation of synthetic indexes of outpatient mental health care in Andalusia (Spain). Epidemiol Psichiatr Soc. 2008 Sep;17(3):192–200. | Not focused on the three dimensions of service delivery |
| 41. | Tomita A, Vandormael AM, Cuadros D, Slotow R, Tanser F, Burns JK. Proximity to healthcare clinic and depression risk in South Africa: geospatial evidence from a nationally representative longitudinal study. Soc Psychiatry Psychiatr Epidemiol. 2017 Aug;52(8):1023–30. | Does not focus on mental health |
| 42. | Fortney. The impact of geographic accessibility on the intensity and quality of depression treatment. 1999 | Not a peer-reviewed research paper |
| 43. | Winckler BE, Nguyen M, Rhee K, Khare M, Patel A, Crandal B, et al. 45.1 Geographic Patterns of Mental Health Utilization in San Diego County. Journal of the American Academy of Child & Adolescent Psychiatry. 2021 Oct;60(10):S240–1. | Not a peer-reviewed research paper |
| 44. | Rine. C. Neighborhood mismatch: Examining mental health service access through proximal distance. 2008 | Not a peer-reviewed research paper |
| 45. | APhA2017 abstracts of contributed papers. Journal of the American Pharmacists Association. 2017 May;57(3):e1–142. | Not a peer-reviewed research paper |
| 46. | Tomlinson M. An examination of the social and community context of substance use disorder recovery support services in Rutherford County, Tennessee. 2021 | Not a peer-reviewed research paper |
| 47. | Edgcomb J, Tseng C hong, Klomhaus AM, Seroussi A, Heldt JP, Perez L, et al. 5.29 First Do No Harm: Measuring Disparities in Child Involuntary Mental Health Detainment Order Use and Provider Justification Using Natural Language Processing. Journal of the American Academy of Child & Adolescent Psychiatry. 2023 Oct;62(10):S268. | Not a peer-reviewed research paper |
| 48. | Isaranuwatchai W. Patterns, determinants, and spatial analysis of health service utilization following the 2004 tsunami in Thailand. 2012 | Not a peer-reviewed research paper |
| 49. | Paper Abstract. J American Geriatrics Society [Internet]. 2018 Apr [cited 2025 Oct 10];66(S2). Available from: <https://agsjournals.onlinelibrary.wiley.com/doi/10.1111/jgs.15376> | Not a peer-reviewed research paper |
| 50. | SAEM Annual Meeting Abstracts. Academic Emergency Medicine [Internet]. 2017 May [cited 2025 Oct 10];24(S1). Available from: <https://onlinelibrary.wiley.com/doi/10.1111/acem.13203> | Not a peer-reviewed research paper |
| 51. | Zacharopoulou Vassiliki, Zarakovitis Dimitrios, Zacharopoulou Georgia, Tsaloukidis Nikolaos, Lazakidou Athina. Geographic Mapping of Use and Knowledge of the Existence of Projects or ICT-Based Devices in Dementia Care. In: Studies in Health Technology and Informatics [Internet]. IOS Press; 2017 | Not a peer-reviewed research paper |
| 52. | 2017 CALDAR Summer Institute and International Conference Promoting Global Health. J Neuroimmune Pharmacol. 2017 Jun;12(S2):81–113. | Not a peer-reviewed research paper |
| 53. | Koziarski. The spatial concentration, stability, and specialization of mental health calls for service: Evidence in support of proactive, place-based interventions. 2023 | Not a peer-reviewed research paper |
| 54. | Friesen E L. Understanding rural-urban disparities in alcohol-related health service use in Ontario, Canada. 2023 | Not a peer-reviewed research paper |
| 55. | Fujita AW, Loughry N, Moore D, Colasanti J, Sheth AN. 1026. What Proportion of Infectious Diseases Physicians Have Buprenorphine Waivers to Treat Opioid Use Disorder in the United States? Open Forum Infectious Diseases. 2022 Dec 15;9(Supplement_2):ofac492.867. | Not a peer-reviewed research paper |
| 56. | Kyungsoo S. The Effects of Acculturation, Health, Socioeconomic Status, and Perceived Respect on Older Adult Depression: Analysis of Korean American Older Adults in Chicago . 2023 | Not a peer-reviewed research paper |
| 57. | Brooks, E. Understanding veterans' service options and utilization patterns for PTSD and TBI. 2011 | Not a peer-reviewed research paper |
| 58. | Abraham PJ, Crowley BM, Moore D, Stephens SW, Minor M, Griffin RL, et al. Understanding the geography of trauma: Combining spatial analysis and funnel plots to create comprehensive spatial injury profiles. J Trauma Acute Care Surg. 2022 Aug;93(2):238–46. | Not a peer-reviewed research paper |
